# 

**Published:** 1856-10

**Authors:** 


### CONTENTS.

ORIGINAL COMMUNICATIONS. Page.

Influence of Mind upon the Physical System, ..... 321
Report on Delirium Tremens, ..... 328
Case of complete Ossification of the Fœtal Head, ..... 344

SELECTIONS.

Report on Quackery, ..... 345
Spontaneous Expulsion in Placental Presentations, ..... 352
Successful Termination in a case of Normal Pregnancy, ..... 361
Rules for Restoring the Drowned, ..... 363
The Laws of Epidemics, ..... 364
The Abnormal Conditions of the Kidneys in Bright's Disease, ..... 366
Perchloride of Iron as an Hœmostatic, ..... 384
Editorial, ..... 383
Bibliographical Notices, ..... 392

